# Specific Learning Disorders and Eating Disorders: an Italian retrospective study

**DOI:** 10.1186/s13052-022-01289-4

**Published:** 2022-06-14

**Authors:** Salvatore Ferdinando Aruta, Jacopo Pruccoli, Nicole Bandini, Paola Rucci, Antonia Parmeggiani

**Affiliations:** 1 IRCCS Istituto delle Scienze Neurologiche di Bologna, Centro Regionale per i Disturbi della Nutrizione e dell’Alimentazione in Età Evolutiva, UO di Neuropsichiatria dell’Età Pediatrica, Bologna, Italy; 2 Dipartimento di Scienze Mediche e Chirurgiche, Università di Bologna, Via Massarenti 9, 40138 Bologna, Italy; 3 Dipartimento di Scienze Biomediche e Neuromotorie, Università di Bologna, Bologna, Italy

**Keywords:** Eating disorders, Neurodevelopmental disorders, Specific learning disorders, Childhood, Adolescence

## Abstract

**Background:**

Although Anorexia Nervosa (AN) patients show dysfunctional behaviour in information processing, visual and verbal memory performance, and different cognitive fields, regardless of their BMI, the literature on the correlations between Eating Disorders (ED) and Neurodevelopmental Disorders (NDD) does not provide conclusive data. Rather than a consequence of the mental disorder, cognitive dysfunctions may be a risk factor for AN.

**Methods:**

Our retrospective study investigates the prevalence of Specific Learning Disorder (SLD) among patients with ED. We considered 262 patients being treated at the Emilia Romagna Feeding and Eating Disorders Outpatient Service in Bologna, Italy. We compared the results with the Italian reference values, according to the most recent data provided by the Italian Ministry of Education.

**Results:**

We found that 25 patients out of 262 (9.54%) presented a comorbid diagnosis of SLD. This SLD prevalence is higher than the Italian reference values (4.9% in the school year 2018/19, *p* < 0.001). Comorbidity with SLD was significantly more frequent in males. A diagnosis of SLD was not associated with a higher frequency of any specific ED diagnosis or with psychiatric comorbidity in general. Positive family history for SLD was not significantly associated with either a positive family history for ED or a diagnosis of SLD.

**Conclusions:**

This is the first Italian study to investigate the prevalence of SLD in ED patients during childhood and adolescence. Our data support previous research documenting that neuropsychological deficit could lead to the development of ED.

## Background

The scientific literature does not provide conclusive data on the correlations between Eating Disorders (ED) and Neurodevelopmental Disorders (NDD). Different neuroscience research centres have recently been trying to characterize a spectrum of cognitive dysfunctions in ED. For example, in 2017 Chen and collaborators proposed an interesting neurophysiological model, which they describe as a “core eating network” model [1]. This network consists of two main pathways – the dorsal control pathway and the ventral reward pathway. According to Chen and collaborators, the balance between these two pathways is a key factor in food intake management. In line with this theory, the ED spectrum could be the result of executive function impairments, ranging from the over-control/rigidness of Anorexia Nervosa (AN) to the under-control/disinhibition of Binge Eating Disorder (BED). Executive functions in ED have also been examined in other relevant studies [2, 3], which describe different test performances in the various ED [4].

Focusing on the cognitive aspects of the single ED, AN seems to have an impact on central coherence, which could explain patients’ typically rigid focus on body details while they overlook global health conditions [5]. Cognitive flexibility and decision-making also appear to be affected [6], possibly impaired by a defective equilibrium between the ventral limbic and dorsal executive circuit activities. These two circuits are involved in wide cognitive functioning such as decision-making and the inhibitory control and reward systems [7]. Dysfunctions in this cognitive functioning could explain such typical aspects of AN as the pathological relationship between food, inhibition, and reward. Consistent with these studies, relevant research on AN patients has evidenced dysfunctions in different cognitive fields unrelated to their Body Mass Index (BMI) [8]. According to these findings, AN patients show dysfunctional behaviour in information processing and memory performance, especially working, visual and verbal memory. The authors concluded that neuropsychological impairments in AN may not be a consequence of the mental disorder; instead, cognitive dysfunctions may be a risk factor for AN. Comparing the various effects of ED on cognitive performances, it appears that compared with Bulimia Nervosa (BN) patients and healthy controls (HCs), AN patients perform poorer executive functions [4].

On the other hand, BN patients exhibit deficient inhibition controls [9] and poorer decision-making ability when compared to HCs [10]. A functional Magnetic Resonance Imaging (fMRI) study compared selected brain activities in BN patients and HCs [11]. BN patients showed a hyperactivation of the parieto-occipital regions and a reduced deactivation of default-mode-network (DMN) areas during alerting.

These dysfunctions correlated with the severity of the ED and were thought to be connected with the typical food and body-shape concerns. Furthermore, compared to HCs, BN patients presented a weaker activity of the anterior cingulate regions, the parahippocampus, and the temporo-parietal junction, during specific attention tasks (i.e., reorienting and executive control of attention) [11]. This hypoactivity could explain other typical BN features such as impulsive behaviour, inattention, and dysfunctional emotion regulation [11].

With regard to the cognitive performances of Binge Eating Disorder (BED) patients, these appear to be in line with AN patients’ results as examinations show a deficiency in central coherence, decision-making, and cognitive flexibility. Decision-making and cognitive flexibility in BED appear even more damaged than in AN patients [6]. Typically, in BED patients, the reward system appears dysfunctional [12]. This has been demonstrated in an MRI study, that focused on rewarding system networks in both the anticipatory and outcome phases. The MRI shows that there is inefficient recruitment of the ventral striatum and the inferior frontal gyrus during the anticipatory phase, whereas there is a decreased activity of the medial prefrontal cortex in the outcome phase [13]. Furthermore, the corticostriatal circuits also appear dysfunctional, thus contributing to the motivation/reward and impulsivity dysfunctions [14].

BED is frequently associated with overweight (OW) or obesity (OB) [12]. Different neurocognitive patterns have been found in OW/OB patients related to the presence/absence of BED. The presence of BED in OW/OB patients is associated with weaker working memory, worse self-regulatory control, and reduced planning skills [15]. Furthermore, BED OW/OB patients appear to be more vulnerable to food-related impulsivity [16].

MRI shows that BED OW/OB patients, when exposed to visual/auditory high-calorie food cues, present a stronger activity of the dorsal anterior cingulate cortex and a weaker function of the prefrontal cortex, probably related to the loss of control over the feeding choice [16, 17].

Following these neurodevelopmental models, scientific research is trying to address the substantial comorbidities between ED and either mental or behavioural disorders, such as Mood Disorders, Anxiety Disorders, Obsessive–Compulsive Disorder, Personality Disorders, Autism Spectrum Disorder, Complex Refusal Syndromes [12, 18–22]. In 2019, Wentz and collaborators published a paper in which they hypothesized a common neurodevelopmental origin in ED and different psychiatric conditions [23].

In Specific Learning Disorders (SLD), a neurobiological origin of the neuropsychological deficits has been repeatedly argued for, even if the cause is still unknown [12]. As mentioned for ED, genetic predisposition could be responsible for altered SLD neurodevelopmental patterns. Studies on twins, which show a common genetic neurodevelopmental root in reading and mathematics ability, support these theories [24].

Furthermore, a comparison of cognitive performances between patients with reading disabilities and patients with math disabilities has shown that both groups manifest common impairment in working memory, processing speed, and verbal comprehension, which supports a multiple-deficit neuropsychological model of SLD [25].

In line with these findings, several research groups have looked for overlaps and psychiatric comorbidities of the most common neurodevelopmental disorders like SLD, Autism Spectrum Disorder (ASD), and Attention-Deficit/Hyperactivity Disorder (ADHD) [26–28].

If the literature concerning ED and general neurodevelopmental disorders is not extensive, scientific publications on ED and SLD are almost non-existent. Our Italian study, which aims to assess the prevalence of SLD in an ED population of children and adolescents, is a first step toward filling a significant gap in this area.

## Methods

### Design

The present paper investigates the prevalence of SLD among ED patients of developmental age. The study is a retrospective chart review involving 262 patients being treated at the Emilia Romagna Feeding and Eating Disorders in children and adolescents Outpatient Service of the IRCCS Institute of Neurological Sciences in Bologna, Italy.

The study aimed to compare the prevalence of SLD among patients with ED in our sample with Italian official SLD reference values [29, 30]. To do so, the inclusion criteria for our study matched the inclusion criteria adopted in the latest document published by the Italian Ministry of Education, which considers patients from the end of the second year of primary school to the last year of secondary school. Early and late SLD clinical manifestations can be detected in several stages of a child’s development; however, according to Italian guidelines, an SLD diagnosis can be made only after the end of the second year of primary school. In compliance with these regulations, we considered patients with a diagnosis of Feeding and Eating Disorders (according to the Diagnostic and Statistical Manual of Mental Disorders, fifth edition), who had completed their second year of primary education and had not completed their last year of secondary education.

The enrollment period was from January 2013 to December 2019. Patients missing proper school documentation/certification and lacking a clear developmental history were excluded from the study. We also did not consider those patients who could not undergo a complete clinical evaluation. Neuropsychiatrists, clinical psychologists, and personnel from our centre performed all diagnostic tests for ED. Clinical psychologists and child neuropsychiatrists specialized in Learning Disorders made the diagnosis of SLD.

### Measures

Demographic characteristics included age, gender, and school year. Family medical history was reviewed searching for ED and SLD. Personal medical history was reviewed searching for a diagnosis of SLD. SLD diagnoses and SLD specifiers were coded according to the Diagnostic and Statistical Manual of Mental Disorders, fifth edition (DSM-5) criteria. SLD specifiers were considered, particularly impairment in reading/dyslexia (DSM-5: 315.00, International Classification of Diseases, eleventh edition (ICD-11): 6A03.0), impairment in written expression (315.2, 6A03.1), and impairment in mathematics/dyscalculia (315.1, 6A03.1). The diagnosis of SLD in Italy requires standardized neuropsychological tests, assessing the cognitive level and learning abilities. As for the cognitive level, an Intelligence Quotient (IQ) equal to or greater than 85 represents a necessary criterion for a SLD diagnosis, obtained preferably with multicomponent scales (e.g., Wechsler Scales). IQ scores lower than 85 obtained with a non-verbal monocomponent intelligence scale, such as Leiter scales or Raven’s Progressive Matrices, need to be confirmed by verbal subtests of multicomponent tests (e.g., Wechsler Intelligence Scale for Children). As for learning abilities, a diagnosis of SLD in Italy requires the assessment of 3 specific parameters: 1) impairment in reading/dyslexia: reading accuracy below the 5^th^ centile and reading speed below 2 standard deviations (SD) from mean standard values for the school year. This diagnosis requires that patients have completed their second year of primary school; 2) impairment in written expression: writing accuracy below the 5^th^ centile (dysorthography) or writing fluency below 2 SD for the school year, with a qualitative analysis of written production (dysgraphia). This diagnosis requires patients’ completion of the second year of primary school; 3) impairment in mathematics/dyscalculia: accuracy and speed of mathematic abilities below 2 SD for the school year. This diagnosis requires patients’ completion of the third year of primary school. When impairment of learning abilities and an IQ ≥ 85 have been demonstrated, a diagnosis of SLD requires the clinical exclusion of sensorial, neurological cognitive, and psychopathological abnormalities as responsible for the documented learning impairment. These diagnostic criteria are recommended in specific government-issued guidelines on the diagnosis of SLD [31–33] and are consistent with the criteria adopted in the identification of the Italian official reference values for SLD [29].

Feeding and Eating Disorders were recorded according to the DSM-5 classification; they included AN, BN, BED, Avoidant/Restrictive Food Intake Disorder, Pica, Rumination Disorder, Other Specified Feeding or Eating Disorder, and Unspecified Feeding or Eating Disorder. Comorbidity with other mental disorders was assessed. Comorbidity with neurodevelopmental disorders (Autism Spectrum Disorder, tic disorders) was reported, although it was not systematically assessed. Clinical data were considered and recorded at the time of the diagnosis for ED in our centre.

### Ethical considerations.

The study has been approved by the Ethical Committee of Bologna (NPI-DA/DSA-2020). Written informed consents were obtained from the patients and their caregivers.

### Statistical analysis

All statistical analyses were performed using JASP, version 0.14.1 for Windows and Stata, version 17.0. Shapiro–Wilk’s and Levene’s tests were used to assess normality of data distribution and homogeneity of variance. Descriptive analyses of demographic and clinical characteristics were performed. Categorical variables were reported as absolute and percentage frequencies; continuous variables were reported as mean ± SD. Patients with and without SLD were compared for demographic and clinical characteristics. Subgroups of patients with different specifiers for SLD (impairment in reading, written expression, and mathematics) were compared as well. Chi-square test was used to compare categorical variables among groups; Fisher’s exact test was adopted when appropriate. Bonferroni correction for multiple comparisons of measures belonging to the same domain was applied to the probability level. Since patients with different ED diagnoses (e.g., AN and BED) were included, BMI was not compared among different clinical groups. We assessed in ur ED sample the cumulative prevalence of SLD in different school years. These values were calculated as the percentage of patients with a diagnosis of SLD, on the total of ED patients coming to our Centre in that school year. These data were then compared with Italian reference values for each school year using the one-sample chi-square test.

## Results

### Clinical characteristics of the sample

A total of 262 patients were enrolled in the study. Demographic and clinical data are reported in Table 1. As for specific ED diagnoses, AN was found in 215 patients (82.1%); 8 patients (3.1%) suffered from BN, whereas 8 patients (3.1%) were affected by BED. The remaining 31 patients (11.8%) presented an Unspecified Feeding or Eating Disorder (UFED).

**Table 1:** Clinical and demographic characteristics of the sample. Values are reported as total numbers and percentages. Age is reported as mean ± standard deviation

Variable	Value
Female gender	218 (83.2%)
Age	14.7 (± 2.7) years
Family history of SLD	2 (0.8%)
Family history of ED	15 (5.7%)
Major Depressive Disorder	11 (4.2%)
Obsessive–Compulsive Disorder	4 (1.5%)
Borderline Personality Disorder	2 (0.8%)
Autism Spectrum Disorder	1 (0.4%)
Generalized Anxiety Disorder	1 (0.4%)
Bipolar Disorder	1 (0.4%)
Tic Disorder	1 (0.4%)

*Abbreviations*: *SLD* Specific Learning Disorder, *ED* Eating Disorders

In examining patients’ personal clinical records, we found that 25 patients out of 262 (9.54%) presented a comorbid diagnosis of SLD. Table 2 shows data concerning the overall prevalence of SLD across different school years in the patients of our study, who received a diagnosis of ED. These data were compared with official Italian reference values for SLD in the same years and proved to be significantly higher starting from 2015/2016. Notably, our study also included patients diagnosed with ED between September 2019 and December 2019. Since official Italian reference data for SLD for the year 2019/20 were not available at the time when this study was conducted, data for these patients are not compared with Italian reference values for the same period. The gender ratio is not reported for Italian national reference values; as for Emilia Romagna, the region where this study was conducted, the percentages for the school year 2018/19 are 60.8% males, 39.2% females [29].

**Table 2:** School years and prevalence of Specific Learning Disorder. Values are reported as the cumulative prevalence of individuals with SLD on the total of individuals per school year

School years	Prevalence of SLD in our sample	Prevalence of SLD in Italy: reference values [29]	χ^2^ test, *p*
2013/14	0/20 0%	2.1%	0.512
2014/15	1.9% (+ 1.9%)	2.6% (+ 0.5%)	0.746
2015/16	6.7% (+ 4.8%)	3.1% (+ 0.5%)	0.05
2016/17	8.7% (+ 2.0%)	3.6% (+ 0.5%)	0.002
2017/18	8.2% (-0.5%)	4.5% (+ 0.9%)	0.016
2018/19	8.5% (+ 0.3%)	4.9% (+ 0.4%)	0.007
Overall (in December 2019)	9.5% (+ 1.0%)	Not available	

*Abbreviations*: *SLD* Specific Learning Disorders

Concerning single specifiers, impairment in reading/dyslexia was present in 5 patients (1.9% of the total); 3 patients (1.2% of the total) exhibited impairment in written expression, and 6 patients (2.3% of the total) presented impairment in mathematics/dyscalculia. In these cases, 1 patient presented impairment in both reading and writing; 1 patient had impairment in both reading and mathematics. For 13 patients (5.0%) the documented diagnosis of SLD was not associated with a specifier. Italian reference prevalence values for the school year 2018/19 are 3.2% for reading impairment, 1.5% for writing impairment-dysgraphia, 1.7% for writing impairment-dysorthography, and 1.6% for mathematic impairment.

### Differences between clinical subgroups

Comorbidity with SLD was significantly more frequent in males (X^2^ = 7.296; *p* = 0.007). As for patients with different ED diagnoses, a diagnosis of SLD was found in 19/215 patients with AN (8.8%), 0/8 patients with BN (0%), and 0/8 patients with BED (0%), and in 6/31 patients with UFED (19.4%). The frequency of SLD comorbidity did not differ significantly among ED diagnoses (X^2^ = 5.270, *p* = 0.153). Moreover, SLD was unrelated to Major Depressive Disorder (*p* = 1.000), OCD (*p* = 1.000), or psychiatric comorbidities in general (*p* = 1.000). Positive family history of SLD was unrelated to a positive family history of ED (*p* = 0.111) or a diagnosis of SLD (*p* = 0.182).

## Discussion

### Main findings

Our study is the first to investigate the prevalence of SLD in a population of patients with ED in Italy. An analysis of cumulative prevalence across different school years made it possible to compare the SLD + ED prevalence in our patients with the Italian SLD prevalence reference values.

Our findings indicate that the prevalence of SLD among our patients was 9.54%. This figure is higher than the Italian reference value. When considering the most recent data provided by the Italian Ministry of Education, the prevalence of SLD for the school year 2018/19 in our patients with ED (9.02%) is considerably higher than the prevalence for the general Italian children population (4.9%). Notably, the prevalence of SLD both in Italian children and in our population with ED has been increasing recently. This may be due to the recent implementation of a more accurate screening/diagnostic protocol. However, the prevalence of SLD in our patients with ED outnumbered Italian reference values only starting in recent school years, specifically in 2015/16.

### Research implications

Our results are consistent with findings of other studies in the ED field. Dysfunctional neurodevelopmental patterns are thought to be a fertile ground for both new neurodevelopmental disorders [26] and psychiatric disorders [27]. A nationwide cohort study in Sweden has documented an increased prevalence of psychiatric and neurodevelopmental disorders in patients with reading problems, with a similar risk in their siblings [28].

Clearly, further research is needed to support the association between ED and SLD. A deeper knowledge of the relationship these disorders could prompt clinicians to detect these conditions since their first signs. This could facilitate the development of targeted screening programs, as happened for other known neurodevelopmental comorbidities [12].

If our data were confirmed, new follow-up strategies could be implemented. As a matter of fact, patients with comorbidities have more often worse psychopathological trajectories, with consequent prognosis implications [12].

Finally, speculations about pathophysiology theories could be put forth. Evidence indicates that exposure to environmental risk factors such as life traumas and social and cultural pressure [12] may serve as a psychopathological catalyst. A possible, bidirectional link between ED and NDD has been documented by recent studies, reporting that autistic social traits in childhood could represent a risk factor for the development of disordered eating in adolescence [34], as well as an increased risk of developing NDD in children born to mothers with ED [35]. Interestingly, further research has documented that prenatal testosterone of progenitors could be involved in the etiology of both AN and ASD in their offspring [36], while fetuses of AN mothers may present delayed fetal brain responses to auditory stimulation [37]. Despite their preliminary nature, these studies, together with our data, indicate the potential existence of a link between ED and NDD, that should be addressed by further, longitudinal research.

### Study limitations

This study has some limitations. First, it is based on a chart review conducted in a specialized centre for the diagnosis and treatment of ED. Part of the patients’ personal medical history concerning SLD could be missing or lacking. This could explain the occasional lack of SLD data in the records (e.g., it has not been reported the specific SLD in 13 SLD patients out of 25). Second, our study considered patients who were attended to over a 7-year period. Cultural, social, and medical attention to SLD (as well as to ED) has been increasing in the last years in Italy. Thus, prevalence values should be considered as influenced by independent factors rather those linked to patients’ neuropsychological status. To address this problem, however, we compared the data we collected for each school year from our sample with the Italian reference values of the same year. Lastly, the relatively small number of male patients (16.79%) in our sample does not allow to confirm evidence from the literature concerning gender differences in SLDs..

## Conclusions

This is the first Italian study to investigate the prevalence of SLD in a population of young patients with ED. The prevalence of SLD in our sample is higher than that documented by Italian reference values, especially for more recent school years. This evidence appears to support previous research documenting that neuropsychological deficit could lead to the development of ED. Further studies are needed to evaluate these findings in broader and clinically selected populations.

## Data Availability

The datasets used and analysed during the current study are available from the corresponding author on reasonable request.

## Abbreviations

AN: Anorexia Nervosa
BED: Binge Eating Disorder
BMI: Body Mass Index
BN: Bulimia Nervosa
DSM-5: Diagnostic and Statistical Manual of Mental Disorders, fifth edition
HCs: Healthy controls
ICD: International Classification of Diseases
ED: Eating Disorders
NDD: Neurodevelopmental Disorders
OCD: Obsessive–Compulsive Disorder
SLD: Specific Learning Disorders
